# Improved Outcomes and Cost with Palliative Care in the Emergency Department: Case-Control Study

**DOI:** 10.5811/westjem.35388

**Published:** 2025-07-11

**Authors:** Brandon Chalfin, Spencer M. Salazar, Regina Laico, Susan Hughes, Patrick J. Macmillan

**Affiliations:** * University of California San Francisco Fresno, Department of Emergency Medicine, Fresno, California; † University of California San Francisco Fresno, Department of Hospice and Palliative Medicine, Fresno, California; ‡ Hawaii Emergency Physicians Associated, Department of Emergency Medicine, Kailua, Hawaii; § University of California San Francisco Fresno, Department of Family and Community Medicine, Fresno, California; || University of California San Francisco Fresno, Department of Internal Medicine, Fresno, California

## Abstract

**Introduction:**

Palliative care consultation teams provide significant advantages for patients, healthcare professionals, and hospitals, particularly in pain management, family support, and clinician satisfaction. Numerous studies show that inpatient palliative care services yield benefits regardless of the timing of initiation, contributing to shortened hospital stays and cost savings. Recent studies have focused on the timing and setting of palliative care, especially in emergency departments (ED), highlighting improved patient outcomes when initiated early. This study explores the potential of embedding hybrid physicians (double-boarded physicians in palliative and emergency medicine) in the ED to further enhance patient care and reduce hospital resources.

**Methods:**

This small pilot case-control study included a subset of all patients referred by emergency physicians and hospitalists for palliative care within 24 hours of registration, physically present in the ED. Cases consisted of all the patients seen by hybrid physicians embedded in the ED. Matched controls were seen by palliative care-boarded clinicians (various other primary specialties) during palliative care rounds in the hospital. Matches were based on diagnosis, comorbidities, and referral date. Outcomes measured included hospital length of stay, total charges, discharge disposition, code status changes, and ED visits not resulting in admission. Statistical analyses used chi-square tests for categorical data and Wilcoxon rank-sum test for continuous data.

**Results:**

In a four-year period, 68 cases were attended by hybrid physicians over 57 disparate days. These cases had significantly shorter hospital stays (median 2.1 days) compared to controls (6.5 days, *P*<.001). Total charges were also lower for cases ($37,800) than for controls ($78,000, *P*<.001). A notable secondary outcome was that 26.5% of ED visits in the case group did not result in hospital admission, compared to all controls being admitted (*P*<.001). In addition, more cases than controls had a code status of comfort care at discharge (*P*=.07)

**Conclusion:**

Embedding hybrid physicians in the ED significantly shortened hospital stays and reduced charges for seriously ill patients. These findings support the further exploration of integrating such physicians into ED settings to enhance patient care and optimize hospital resources.

## INTRODUCTION

The advantages of incorporating palliative care consultation teams for patients, clinicians, and hospitals have been extensively documented, particularly in areas such as improving pain management, bolstering family support, and enhancing clinician satisfaction.^1^ Research conducted over the past two decades has consistently demonstrated the positive impact of inpatient palliative care services, irrespective of the timing of their initiation. These benefits have been observed across various complex metrics, including studies conducted on a national population scale, such as in Canada,^2^ as well as within community healthcare settings.^3^

While acknowledging the inherent value of inpatient palliative care services as an initial point of intervention, recent studies have underscored the critical role of timing and the clinical setting in which consultations take place.^4^ These findings counter a previous review from 2016 that had suggested inconclusive benefits associated with palliative care services initiated in the emergency department (ED).^5^ In contrast, a 2019 review argued that existing data supports the feasibility and potential quality-of-life improvements associated with palliative care in the ED setting, without apparent adverse effects on patient survival.^6^

Furthermore, studies have consistently shown that early initiation of palliative care services can yield substantial cost reductions, shorter hospital stays,^7^ and lower readmission rates.^8^ For example, while initiating inpatient consultations within three days has demonstrated clear benefits, a retrospective analysis revealed that ED-initiated consultations were associated with significantly shorter lengths of stay (LOS) for hospitalized patients. This aligns with the patient- and family-centered benefits of palliative care and contributes to reduced inpatient resource utilization.^9^ A recent study by Macmillan et al even showed that consultations initiated within 24 hours were significantly associated with reduced LOS and lower hospital charges, regardless of the underlying disease.^10^ It is worth noting the downstream effects on inpatient services, particularly since rapid response team calls often relate to end-of-life symptoms.^11^

Williams et al emphasized the necessity for earlier palliative referrals and highlighted the potential to identify patients at high risk of in-hospital mortality upon admission, using the Criteria for Screening and Triaging to Appropriate Alternative Care (CriSTAL) criteria tool to encourage prompt palliative referrals.^11^ The CriSTAL is a tool designed to identify elderly patients nearing the end of life. Developed using objective criteria derived from existing scales and research, CriSTAL is one of several screening tools available to help determine patients who may benefit from goals-of-care discussions. Admission triggers have already demonstrated their benefits, with criteria such as end-stage illness, functional limitations, and clinician anticipation of in-hospital death.^7^ Consequently, it is reasonable to hypothesize that rapid palliative interventions hold similar value.

**Population Health Research Capsule**

What do we already know about this issue?

*Palliative care in the ED improves patient outcomes, reduces hospital stays and costs, and enhances clinician satisfaction, especially when initiated early*.

What was the research question?

*Does embedding hybrid palliative-emergency physicians in the ED improve patient outcomes and reduce costs?*

What was the major inding of the study?

*Palliative care cases had shorter hospital stays (median 2.1 days) compared to controls (6.5 days, P<0.001), and lower total charges ($37,800 vs. $78,000, P<.001). Admissions were avoided in 26.5% of ED visits with palliative care vs. 0% of controls*.

How does this improve population health?

*Embedding palliative care in the ED enhances early goals-of-care discussions, reduces unnecessary admissions, lowers costs, and improves end-of-life care quality*.

The integration of palliative care into EDs addresses a significant need, as many patients present without advance directives or established goals of care. A systematic review published found that only 36.7% of US adults had completed an advance directive, with 29.3% having living wills. Notably, the completion rates were similar between patients with chronic illnesses (38.2%) and healthy adults (32.7%), indicating that a significant proportion of individuals with serious health conditions lack documented end-of-life care preferences.^12^ Studies also indicate that a substantial proportion of older adults, 56%–99%, do not have advance directives available upon ED admission.^13^ Additionally, research has shown that among patients who underwent cardiopulmonary resuscitation in a respiratory care unit, 59% died within 24 hours, highlighting the critical importance of timely palliative interventions.^14^ Furthermore, a second systematic review found that approximately 17% of patients visiting the ED had a terminal illness, emphasizing the ED’s pivotal role in initiating palliative care discussions.^15^ These findings underscore the necessity for early palliative care involvement in the ED to facilitate discussions on goals of care and advance directives, ultimately improving patient outcomes and ensuring that treatment aligns with patient preferences.

Collectively, the studies above illustrate the superiority of initiating consultations within 24 hours and within the ED setting. Given the proven advantages of having palliative-minded ED personnel and effective tools for identifying palliative-appropriate patients as early as possible, the logical next step would be to explore the potential benefits of having hybrid physicians (dual-boarded in palliative and emergency medicine [EM]) in the ED available for immediate consultation. The number of patients presenting to the ED daily, whether due to hospital readmissions, preexisting hospice enrollment, or catastrophic injuries, further supports embedding palliative medicine within this setting. Having EM-trained physicians with specialized palliative expertise is a strategic approach to enhancing the healthcare system’s overall function. The earlier we engage patients and families in critical goals-of-care discussions, the greater the potential to improve the value and impact of these conversations. In this hybrid physician case-control study we aimed to assess the effectiveness of deploying hybrid physicians onsite to evaluate patients referred for consultation in the ED, comparing these encounters to referrals made by emergency physicians (EP) to the current standard of inpatient palliative care teams.

## METHODS

We conducted this investigation at a community-based, urban, tertiary-care hospital in central California, which is affiliated with a local university and has ≈700 beds. The hospital’s inpatient palliative care team is responsible for handling consultations across various hospital departments, including the ED, intensive care units (ICU), and general inpatient floors. The Community Health System Institutional Review Board approved this study as exempt under 45 CFR 46, and patient consent was waived under 45 CFR 46.116 (d).

### Study Design

For this case-control study, we collected and analyzed records from the palliative care department encompassing referrals and consultations made between August 2018–December 2022. Consults at our hospital are need-based with reasons for a consult including the following: goals-of care-discussions; hospice patients; advance care planning; comfort care patients; pain and symptom management; and frequent ED visits or hospitalizations. The palliative care department at our hospital conducted an average of 2,360 consults a year during the study, for an average of 6.5 consults per day. As this was a pilot project, our double-boarded physicians were only working in this hybrid role on limited days because the hybrid physicians were working as EPs as well as being scheduled on the general inpatient palliative service. This role was only possible when all our inpatient palliative teams were fully staffed and allowed for an additional hybrid physician to be embedded in the ED to collect cases. Furthermore, during the pandemic, fewer days were possible because of alternative needs of the palliative department across the hospital on the inpatient service. We categorized patients into two groups:

#### Cases

These were patients who received a palliative care referral from an emergency clinician within 24 hours of their initial hospital registration and were attended to by a hybrid physician embedded in the ED (available to respond urgently during scheduled palliative shifts in the ED). Cases were all seen by the hybrid physicians during the study period

#### Controls

Controls were selected on a one-to-one basis from a randomized list of patients who received referrals within 24 hours of their initial hospital registration by an EP while the patient was still in the ED; but these patients were attended to by a palliative care clinician who did not specialize in EM during routine palliative care rounds.

To mitigate bias, the selection of controls was based on three variables: underlying diagnosis; comorbidities; and the date of referral. Specifically, we considered the patient’s underlying medical condition, distinguishing it from the billable diagnosis, which is a standard practice carried out by palliative care team members. Additionally, we used the Charlson Comorbidity Index (CCI), a validated tool for assessing disease burden. This index assigns scores on a scale from 0–36, with age factoring into the score, whereby older patients accrue more points. Controls were matched to cases by ensuring that the CCI scores were within three points of each other.

We also matched controls to cases using the dates of the referrals, ensuring that they occurred within a three-month window (either before or after the case referral). The data used for matching cases to controls was derived from palliative care records and included information such as underlying disease (categorized by malignancy type or chronic/terminal illness), admission and discharge dates and times, and the date and time the palliative care order was placed. Data needed to calculate the CCI scores were obtained through a review of patient charts.

### Outcome Measures

In our analysis we considered potential patient-specific confounders, such as age, sex, and race/ethnicity, which were sourced from palliative care records. We also assessed potential differences in the treatment of cases and controls, considering discharge disposition, code status changes, code status at discharge, physician orders for end-of-life treatment, and any ED visits that did not result in hospital admission as a secondary outcome measure. The primary outcome variables under investigation included the length of hospital stay and total charges incurred over the course of care.

### Statistical Analysis

We used chi-square tests to compare categorical data to test for differences between cases and controls. Non-parametric Wilcoxon rank-sum tests were used to compare continuous data. We tested matching variables to check on effectiveness of the match process. Basic demographics that were not part of the match were tested for differences between cases and controls. A two-sided *P*-value of less than .05 was considered statistically significant. We used SAS software v 9.4 (SAS Institute Inc, Cary, NC) for all analyses.

## RESULTS

In the four years and four months we conducted this study, a total of 68 cases were seen over 57 days, averaging 1.2 consults per day. On days that the hybrid physicians were working in this role, they saw 18.5% of the total consults for that day. Table 1 shows the underlying diseases for the cases and matched controls. Non-hematologic cancer was 37% of the sample with dementia the next highest at 21%. We were able to find controls that matched all the cases. The matching variables of CCI score and date of referral were not significantly different (median, *P*-value: CCI 0, .93; referral date −27; .06).

Table 2 shows the demographic characteristics of the cases and controls. Of note, only race/ethnicity was significantly different with more White patients as cases and more Hispanic patients as controls (*P* < .01).

Table 3 contains the outcomes we tested. Of note, we found significant differences in our primary outcomes of length of hospital stay and total charges. The median hospital LOS for cases was 2.1 days (Q1 – Q4; 0.5 – 5.1) while controls stayed 6.5 days (Q1 – Q4; 4.2 – 12.2; *P* < .001). The median total charge for cases was $37,800 (Q1 – Q4; $15,200 – $67,800), while the controls median total charge was $78,000 (Q1 – Q4; $34,600 – $135,900; *P* < .001). One secondary outcome was significant; 26.5% of ED visits that did not have an hospital admission occurred in the case group and were seen by the hybrid physician while all controls were admitted to the hospital (*P* < .001). In addition, more cases than controls had a code status of comfort care at discharge (*P* = .07). Patients who changed code status after palliative care consultation overwhelmingly changed to comfort care.

## DISCUSSION

The uptick in EPs transitioning into the field of palliative medicine is noteworthy.^16^ We have personally seen more EM-trained physicians apply to our hospice and palliative medicine (HPM) fellowship and have trained three EPs as of January 2025. In our training program we have two EM-trained physicians on our HPM faculty. One of those physicians was hired specifically to look at the effectiveness of placing them in the ED. We know there is an association with decreasing LOS and hospital charges the faster our palliative care team sees patients,^10^ but what would the impact be if that team or a physician was embedded in the department where there are established relationships and a chance to see patients even sooner?

The number of patients that end up in the ED daily, whether it is hospital readmissions, patients already on hospice, or catastrophic injuries, warrants that palliative medicine physicians embed themselves in the ED.^10^ Having EM-trained physicians who obtained a specialty in palliative medicine seems prudent to the overall functioning of the healthcare system. The further upstream we encounter patients and families to have critical goals-of-care discussions the better we can impact the value of these discussions.

Our study showed that among patients with a variety of underlying diseases who were seen by our hybrid physician, there was a median reduction in LOS and total hospital charges. Patients seen by the hybrid physician stayed in the hospital four fewer days than controls. In both cases and controls, when a code status was changed, it was overwhelmingly changed to comfort care (88.1% and 86.1%, respectively), illustrating the impact that palliative care consultation can have in general. The interventional group also saw a reduction in total hospital charges by approximately $40,000. Of the cases seen by our hybrid physician, 26.5% were not admitted to the hospital compared to the controls, who were all admitted. The patients who were not admitted could have been discharged somewhere such as home, with hospice, or died under the care of the EP without admission. Avoiding unnecessary admissions and shortening LOS, thereby resulting in decreased resource utilization, were the goals of this pilot study. Additionally, more cases from the study group were transitioned to comfort care and had a higher mortality. This is an important finding since transitioning to comfort care often leads to shortening the LOS and resource utilization as they are typically moved into hospice care outside the hospital.

Transitioning to comfort care is for patients who have essentially been transitioned to hospice in the hospital and are receiving symptom-directed care that focuses on comfort and no aggressive or life-sustaining interventions. This avoids expensive studies and investigations that often lead to more suffering for patients and decreases high-value care. Another finding is that the cases had fewer hospice consults compared to the controls, although this difference was not statistically significant and was not a primary focus of our study. One possible explanation is that patients who prompted earlier palliative involvement in the ED by the hybrid physician may have been less stable for a transition to outpatient hospice. Instead, these patients may have transitioned to comfort measures and died in the hospital. These findings align with a recent study on embedding palliative care in an ED, emphasizing early integration of palliative care to improve patient outcomes and resource utilization.^17^ The study reported a significant increase in ED consultations, high satisfaction among clinicians and nurses, reduced hospitalization and costs, and a notable 6.7 times return on investment.^17^ Embedding palliative care in the ED was found to streamline workflows and improve patient care, suggesting its potential as a model for enhancing care for seriously ill patients in other healthcare systems.^17^

Two of our hybrid physicians were involved in direct patient encounters. Understanding the complexities of the ED setting makes them better equipped to handle complex end-of-life discussions in this environment, theoretically. These hybrid physicians are well versed in the operations of both the ED and the palliative care department. Because the hybrid physicians are attending physicians in EM, they have professional relationships with all their EM colleagues enabling a more seamless collaboration. Lastly, by being available, hybrid physicians were able to deal with complex urgent palliative needs such as goals-of-care discussions that arose in the ED. Specifically, urgent goals-of-care conversations with patients with terminal diagnoses that otherwise would have been intubated and admitted to the ICU could occur. These urgent goals-of-care conversations could result in a transition to comfort measures and subsequent in-hospital death.

From our data, we conclude that more programs and hospitals should explore embedding dually trained physicians in EM and HPM to work in the ED setting. Additionally, adding a full complement of palliative care staff (registered nurse, chaplain, and social worker) might better meet the needs of patients receiving care in the ED.

## LIMITATIONS

Limitations to this study include basing the study on one hospital system vs multiple hospitals, as well as the low number of patients in our intervention group. However, we believe our methods add robustness even with this low number. Additionally, we did not document acuity at the time of presentation. Instead we included underlying disease and a measure of comorbidity status in our matching criteria. All cases and controls were matched within a ± three-month date of referral to address changes in the system/circumstances over time.

Acquiring cases for the study took a long time due to several factors. We began our study before the COVID-19 pandemic, and staffing issues made it difficult for our faculty emergency/ palliative physicians to work in the hybrid role and gather cases. This role was only feasible when inpatient palliative teams were fully staffed, allowing an additional hybrid physician to be placed in the ED. As a result, emergency/palliative physicians worked in this role for about 57 days. Additionally, many consults originated in the ED from hospitalists, and we aimed to differentiate these from the usual consults we received from the hospitalist group.

During the days that cases were being collected, EPs were informed of the possibility of getting a consult from the hybrid physician if needed. However, they were not informed that a study was taking place. Since referrals are need-based it is possible that bias was introduced by this knowledge.

## CONCLUSION

Our study at a university-affiliated community-based hospital in central California demonstrates that consults for palliative care seen by dually trained palliative/emergency physicians in the emergency department provides a significant association in reduced length of stay and hospital charges in patients regardless of their underlying disease.

## Figures and Tables

**Table 1:** Underlying diseases of cases and matched controls in a study of outcomes of emergency department palliative care physician intervention.

Underlying disease	N	Percentage a
Aneurysm	1	1
Chronic heart failure	5	7
Chronic obstructive pulmonary disease	6	9
Cardiovascular accident	2	3
Cyclic vomiting syndrome	1	1
Cirrhosis	1	1
Debility/frailty	2	3
Dementia	14	21
End-stage renal disease	8	12
Malignancy (hematologic)	3	4
Malignancy (non-hematologic)	25	37
Total cases	68	99

a Percentages do not add up to 100% due to rounding error.

**Table 2:** Demographic characteristics of cases and controls in a study of outcomes of emergency department palliative care physician intervention.

Characteristic	Cases	Controls	P-value
Age, years (median, Q1–Q3)	75 (62.5 – 87)	71 (62 – 81)	.23
Sex (%)			.30
Female	62	53	
Male	38	47	
Race / Ethnicity (%)			<.01
Black	10	13	
Hispanic	26	51	
White	53	26	
Other a	5	6	

Q1 is the value in the 25^th^ percentile; Q3 is the value in the 75^th^ percentile.

a Other race/ethnicity includes American Indian, Asian, East Indian, and Pacific Islander.

**Table 3:** Outcome characteristics of cases and controls in a study of outcomes of emergency department palliative care physician intervention.

Characteristic	Cases	Controls	P-value
Code status at discharge (%)			.07
Comfort care^a^	48.5	29.4	
DNR/DNI	30.9	39.7	
Full code	20.6	30.9	
Code changed (%)			.31
No	38.2	47.1	
Yes	61.8	52.9	
If code changed, was to comfort care b (%)			.80
No	11.9	13.9	
Yes	88.1	86.1	
Discharge status c (%)			.15
Died	29.4	14.7	
Home	29.4	39.7	
Hospice	22.1	26.5	
Skilled nursing facility	16.1	19.1	
Other	2.9	0.0	
Physician orders for life-sustaining treatment (%)			.16
No	57.4	69.1	
Yes	42.6	30.9	
Emergency department visit only (%)			< .001
No	73.5	100.0	
Yes	26.5	0.0	
Emergency department visit after this palliative care consult d (%)			.75
No	58.3	55.2	
Yes	41.7	44.8	
Hospital admission after this palliative care consult d (%)			.79
No	64.6	62.1	
Yes	35.4	37.9	
Hospice consult (%)			.16
No	67.6	55.9	
Yes	32.4	44.1	
Intensive care unit admission (%)			.19
No	86.8	77.9	
Yes	13.2	22.1	
Hospital length of stay, median days (Q1 – Q3)	2.1 (0.5 – 5.1)	6.5 (4.2 – 12.2)	< .001
Total charges, median $1,000 (Q1 – Q3)	37.8 (15.2 – 67.8)	78.0 (34.6 – 135.9)	< .001

Q1 is the value in the 25^th^ percentile; Q3 is the value in the 75^th^ percentile.

a Comfort care refers to patients who have essentially been transitioned to hospice and are receiving goal-directed care that focuses on comfort and no aggressive or life-sustaining treatments.

b Calculated using patients who changed their code status (42 cases and 36 controls).

c Percentages do not add up to 100% due to rounding error.

d Calculated using patients still alive (48 cases and 58 controls).

*DNR/DNI*, do not resuscitate/do not intubate.
